# A Methodological Evaluation of Four Different Paired Associative Stimulation Paradigms in Healthy Controls

**DOI:** 10.3390/brainsci15050461

**Published:** 2025-04-27

**Authors:** Kenan Hodzic, Magnus Thordstein, Joakim Strandberg, Elisabet Jerlhag, Caroline E. Wass

**Affiliations:** 1 Division of Pharmacology, Institute of Neuroscience and Physiology, The Sahlgrenska Academy, University of Gothenburg, 405 30 Gothenburg, Sweden; kenan.hodzic@vgregion.se (K.H.); elisabet.jerlhag@pharm.gu.se (E.J.); 2 Department of Biomedicine and Clinical Sciences, Linköping University, 581 83 Linköping, Sweden; magnus.thordstein@liu.se; 3 Department for Clinical Neurophysiology, Sahlgrenska University Hospital, 413 45 Gothenburg, Sweden; joakim.strandberg@gu.se; 4 Section for Clinical Neuroscience, Institute of Neuroscience and Physiology, The Sahlgrenska Academy, University of Gothenburg, 405 30 Gothenburg, Sweden; 5 Department of Psychiatry for Affective Disorders, Sahlgrenska University Hospital, 413 45 Gothenburg, Sweden

**Keywords:** transcranial magnetic stimulation, paired associative stimulation, plasticity, peripheral nerve stimulation, neurophysiology, psychiatry

## Abstract

**Background/Objectives**: Plasticity deficits play a key role in the pathophysiology of various psychiatric and neurological disorders. Paired associative stimulation (PAS) leverages Hebbian principles to induce synaptic plasticity in the human brain. By repeatedly pairing (1) the peripheral nerve stimulation of the median nerve with (2) transcranial magnetic stimulation over the primary motor cortex (M1) at different inter-stimulus intervals (25 ms; PAS-25, or 10 ms; PAS-10), corticospinal excitability can be increased (PAS-25, mimicking long-term potentiation (LTP)) or decreased (PAS-10, mimicking long-term depression (LTD)). However, variations in the number of pairings and inter-pair intervals lack consensus. The aim of the study was to evaluate four different PAS paradigms, i.e., PAS-10 and PAS-25 with both 180 versus 225 pairings each, to establish the most reliable PAS protocols for LTP- and LTD-like cortical changes. **Methods**: In a randomized, double-blind, crossover study, 14 healthy participants underwent PAS-10 and PAS-25 with 180 and 225 pairings. Excitability was assessed by quantifying the EMG response amplitude of a hand muscle to a single stimulus. **Results**: PAS-25 with 225 pairings produced a robust enhancement of corticospinal excitability, while PAS-25 with 180 pairings was less effective. Surprisingly, PAS-10 with both 180 and 225 pairings also increased excitability. **Conclusions**: While all four PAS paradigms enhanced M1 excitability, PAS-25 with 225 pairings induced the strongest group-level effects and was most time-efficient. Significant individual variability of PAS responses suggests that optimizing PAS parameters, including pairing number and interstimulus intervals, may be necessary for personalized approaches.

## 1. Introduction

Plasticity is the nervous systems’ ability to adapt and re-arrange synaptic connections and reorganize circuitry in response to internal or external stimuli, critical to cognitive, emotional, and behavioral functioning throughout the life span [1,2]. Plasticity encompasses processes such as neurogenesis, neurotrophic signaling, neurotransmitter modulation, and synaptic remodeling. These mechanisms are thought to be disrupted in the pathophysiology of, e.g., depression [3] and schizophrenia [4]. For example, in major depression, there is reduced memory function and hippocampal volume, potentially related to deficient growth factors and neurotrophic factor signaling and subsequent aberrant plasticity. Such impairments in plasticity may be partly restored by electroconvulsive therapy and ketamine treatment [5]. Moreover, in major depressive disorder, deficient connectivity within and between the default mode network and central executive network is thought to contribute to the pathophysiology [6,7]. Non-invasive brain stimulation treatments, such as repetitive transcranial magnetic stimulation (TMS), may counteract these deficits by modulating cortical plasticity, contributing to the antidepressant effect [6,7]. In schizophrenia, deficits in plasticity and cognition impair everyday functioning, and NMDA receptor acting agents as well as TMS-based treatments are proposed to alleviate these deficits [8,9]. Thus, research on brain plasticity has advanced our understanding of the pathophysiology of neurological and psychiatric disorders while driving innovative therapies. Expanding the knowledge of neural plasticity is essential for developing targeted treatments, making it crucial to define methods that effectively induce synaptic plasticity [10].

Paired associative stimulation (PAS) enables the assessment of synaptic plasticity in the human cortex by combining repetitive TMS and peripheral nerve stimulation (PnS) [11]. It involves the close-to-synchronous stimulation of a peripheral nerve, e.g., the right median nerve, paired with TMS stimulation of the corresponding area in the primary motor cortex, within a narrowly defined time window (the inter-stimulus interval). Depending on the inter-stimulus interval between the primary PnS and secondary TMS delivered stimulus, PAS enhances or inhibits corticospinal excitability [12]. Plasticity-induced changes of excitability are assessed by quantifying the EMG response amplitude of a muscle to a single stimulus. These changes are thought to be comparable to Hebbian synaptic plasticity, i.e., long-term potentiation (LTP) and long-term depression (LTD). PAS-induced LTP- and LTD-like responses demonstrate temporal specificity [12]: PAS LTP is induced at PnS-TMS inter-stimulus intervals around 25 ms (PAS-25). PAS LTD is typically induced at inter-stimulus intervals of 10 ms (PAS-10) [13]. These PAS changes rely on neurochemical properties mimicking those observed in in vitro LTP and LTD: dependence on NMDA- [14,15], GABA-B [16], dopamine- [17], and nicotinic [18,19] receptor functioning.

There are large methodological variations in PAS paradigms, particularly concerning the number and frequency of paired stimuli, across research settings. The original PAS study used 90 paired pulses with an inter-stimulus interval of 25 ms (PAS-25) with pairs presented at 0.1 Hz (i.e., pairs presented every 10 s), resulting in a robust increase in the post-PAS EMG response [11]. Altering the interval between pairings influences the protocol length [20], and a meta-analysis confirmed that 0.05 (i.e., pairs presented every 20 s) and 0.2 Hz (i.e., pairs presented every 5 s) inter-pair frequencies produced the most robust PAS LTP effects [21]. It was also demonstrated that seven of eight studies using 225 paired pulses found a significant PAS effect [21]. Other laboratories produced significant PAS-25 effects using 180 paired pulses at 0.1 Hz [9,22,23]. Moreover, delivering a higher number of paired pulses in lower-limb PAS interventions (e.g., 360 pairings instead of 180) tends to enhance cortico-spinal excitability [24]. It remains to be clarified how different stimulation parameters, such as the number of paired pulses and inter-pair intervals, specifically influence cortico-spinal excitability, and the lack of standardization may explain variable outcomes. To address this, the present study aimed to conduct a comparative analysis of four established PAS protocols: PAS-10 and PAS-25 with 180 paired pulses at 0.1 Hz (i.e., pairs presented every 10 s) and PAS-10 and PAS-25 with 225 paired pulses at 0.25 Hz (i.e., pairs presented every 4 s). These paradigms have been used in various studies to explore changes in plasticity to elucidate different neurological and psychiatric pathological mechanisms [25,26,27,28]. This study aimed to deepen the understanding of how distinct stimulation parameters affect neural plasticity.

## 2. Materials and Methods

### 2.1. Study Design

**Figure 1:**
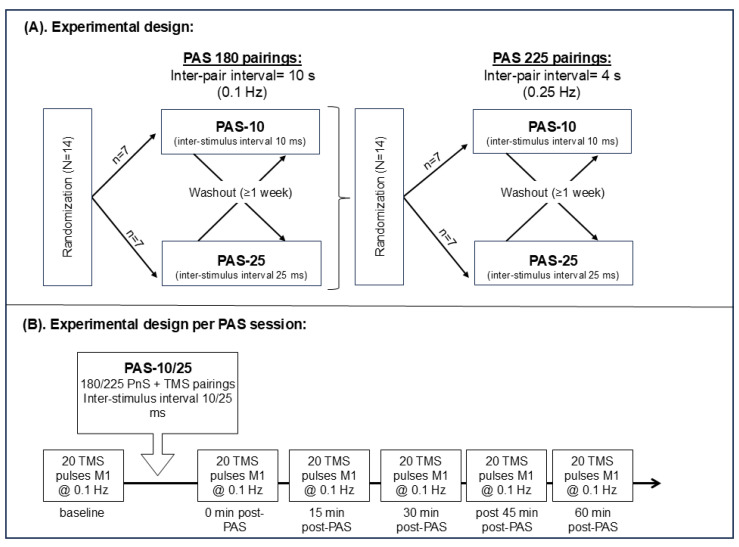
(**A**) Experimental design consisting of two phases: (1) 180 PnS + TMS pairings and (2) of 225 PnS + TMS pairings, 14 healthy participants were randomized to receive either of PAS-10 (n = 7) or PAS-25 (n = 7) first and were after at least 1 week washed out and crossed over to receive the other treatment. (**A**,**B**) The 180 PnS + TMS pair was delivered every 10 s (i.e., @0.1 Hz) for a total of 30 min, while the 225 PnS + TMS pair was delivered every 4 s (i.e., @0.25 Hz) for a total of 15 min. (**B**) Baseline and post-PAS MEPs consisted of 20 single pulses (every 10 s, i.e., @0.1 Hz) targeting the primary motor cortex. Post-PAS MEPs were assessed at 0, 15, 30, 45, and 60 min after PAS. Abbreviations: PAS = paired associative stimulation, PnS = peripheral nerve stimulation, TMS = transcranial magnetic stimulation, and M1 = primary motor cortex.

The study was carried out as a double-blinded (as regards to inter-stimulus intervals), randomized, crossover study consisting of a total of four test sessions, each separated by at least 7 days, see Figure 1A for experimental design. Subjects were randomly assigned to different PAS paradigms using a computerized chance generator such that the experimenter was blind to which paradigm the subject received. Randomization was handled using software written in C++ Visual Studio 2010. The same software controlled the delivery and timing of TMS and PnS pulses based on a randomized subject number.

### 2.2. Participants

Healthy controls were recruited via newspaper ads and advertisement at the University of Gothenburg, Sweden (Approved by the Ethical Review board of Gothenburg, Sweden). Fourteen healthy controls were included in the four PAS testing sessions, evaluating the effects of PAS-10 and PAS-25 using 180 and 225 pairings. Inclusion criteria were as follows: age 18–45 years, psychiatrically healthy (i.e., no DSM-IV axis I diagnosis, with the exception of a depressive episode in remission, ≥6 months), somatically healthy, and right-handedness (assessed by Oldfield Handedness Inventory [29]). Females had to use contraceptives or display a negative urine pregnancy test. Exclusion criteria were as follows: Use of tobacco ≤6 months, high alcohol consumption (AUDIT score ≥ 5 for females and ≥6 for males), use of narcotic drugs ≤ 6 months, and BMI > 27.

### 2.3. Transcranial Magnetic Stimulation:

TMS stimulation was delivered using a figure-of-eight coil, applying biphasic stimulations (duration 280 µs) in a posterior–anterior direction, connected to an eXimia TMS stimulator (Nexstim Ltd., Helsinki, Finland). The coil (outer diameter of each wing, 90 mm) was placed at a 45° angle from the midline of the head and flat against the skull of the subject. Navigated Brain Stimulation software (NBS, software version 3.2.1, Nexstim Ltd., Helsinki, Finland) was used to target and deliver stimulations at desired locations using a standard magnetic resonance image produced by the software. Subjects were fitted with tracking goggles, allowing a stereotactic camera connected to the system to track the subjects’ heads in real time using retroflective materials fitted onto the goggles as well as the coil.

### 2.4. Electromyography

To identify the cortical region corresponding to the abductor pollicis brevis muscle (APB), repeated single-pulse TMS stimulations were delivered to the motor cortex (M1). Each pulse elicited a MEP that was recorded with EMG using Ag–Ag/Cl surface electrodes with a measuring area of 95 mm^2^ (Ambu Neuroline 720, Ambu, Helsingborg, Sweden). The active electrode was placed over the muscle belly, and the reference electrode was placed over the interphalangeal joint of the thumb. A ground electrode was placed on the dorsal side of the hand. The recorded EMG was sampled at 3000 Hz and band-pass filtered (between 10 and 500 Hz).

### 2.5. Peripheral Nerve Stimulation

Electrical stimulation of the median nerve (PnS) of the hand was performed using a surface stimulation electrode at a fixed distance (Nihon Kohden NM-422B, Nihon Kohoden Corporation, Tokyo, Japan). The stimulator was placed over the ventral right forearm at the level of the wrist with the cathode proximally, corresponding to the median nerve, assessed by repeated PnS stimulations and observing muscle activity in the APB. The perceptual threshold for the PnS intensity was determined, with the lowest stimulation intensity perceived by the subject. During PAS, the PnS stimulation (stimulus width, 200 µs) intensity was set to 300% of the perceptual threshold.

### 2.6. Resting Motor Thresholds and PAS Stimulation Intensity

Starting at 50% TMS intensity, the motor cortex hotspot was located as the maximal EMG output. The resting motor threshold (RMT) was determined as the lowest stimulation intensity producing at least 50 µV EMG response in 5 out of 10 stimulations. PAS stimulation intensity was set to 120% of RMT, aiming to produce a response amplitude of ~0.7 to 1 mV peak-to-peak (mean of 20 stimulations).

### 2.7. PAS Testing

The PAS protocols involved repetitive delivery of two paired stimulations: (1) electrical stimulation of the right median nerve, followed by an interstimulus interval of 25 ms (for LTP [11]) and 10 ms (for LTD [15]); (2) a TMS pulse delivered to the left M1, hence PAS-25 and PAS-10. In total, four different PAS protocols were evaluated: (1) PAS-10 (180, [30]); (2) PAS-25 (180, [9]); (3) PAS-10 (225, [31]); (4) PAS-25 (225, [23]), see Table 1 for stimulation parameters.

The evaluation of 180 pairings (both PAS-10 and PAS-25) was carried out in the first phase of experiments, and 225 pairings (both PAS-10 and PAS-25) were evaluated in the second phase of experiments, see Figure 1A.

The PAS-10 or PAS-25 protocols were executed in a random order, determined by the software containing the randomization list. PAS sessions were separated by at least one week, and the two different paradigms (i.e., 180 vs. 225 paired PAS pulses) were tested one year apart due to logistic limitations. Plasticity induction was assessed as changes in TMS-induced MEP amplitudes: mean of 20 stimulations at 0.1 Hz at baseline and 0, 15, 30, 45, and 60 min post-PAS, see Figure 1B.

### 2.8. Attention

To gauge the subjects’ attention, they were instructed to focus their attention and to keep looking at the stimulated hand and recite the number of stimulations received at six predetermined time points during PAS. The difference between the number of stimulations delivered and the amount received was calculated for each participant at each time point, and the total mean of recited errors was calculated for each paradigm.

### 2.9. Statistics

To estimate the sample size required, an approximation in accordance with previous successful PAS studies, where sample sizes varied from nine [27] to 12 [22] healthy subjects, was made. After conducting a post hoc power analysis, the sample size was calculated using G*Power 3.1 for a repeated measure one-way ANOVA (rmANOVA) within subjects’ factors, using the following parameters: effect size f = 0.3 (equivalent of a Cohen’s d = 0.6), power (1 − β) = 0.8, and α = 0.05. This resulted in a final sample size of 14. Normality testing was carried out using the Kolmogorov–Smirnov analysis. Responders were defined by estimating the grand mean of all post-PAS measurements and dividing it by the baseline mean. A quotient > 1.0 was required for PAS-25 and <1.0 for PAS-10.

The primary aim was to assess change in mean MEP, comparing baseline to changes at different time points post-PAS (i.e., 0, 15, 30, 45, and 60 min) for the four different PAS protocols. LTD was defined as a decrease in mean MEP comparing baseline to cumulative post-PAS change in MEP (i.e., mean of post-PAS 0–60 min measures), while LTP was defined as an increase in mean MEP comparing baseline to post-PAS change in MEP (i.e., mean of post-PAS 0–60 min measures). To analyze the change in measured MEP amplitudes over time in each separate protocol, a linear model was applied to execute an rmANOVA, with the within-subjects factor time, in the whole study group, excluding non-responders. For data with a skewed distribution, Friedman’s test was applied for repeated measures, followed by the Wilcoxon post hoc test for paired comparisons of post-PAS time points. Post hoc Bonferroni corrected analysis or Wilcoxon test was performed for pairwise comparison between different time points and baseline when data were normally distributed. Variability testing between sessions was performed for RMT levels and cumulative post/pre ratios per paradigm using rmANOVA.

Relationships between baseline demographics, such as age, height, RMT, stimulus intensity, and PAS response, were analyzed in responders using Pearson and Spearman correlations.

The difference between the number of stimulations delivered and the number recited was calculated for each participant at each time point, and the total mean of the recited errors between sessions for each participant was calculated for each paradigm. The means were then compared between paradigms using the Mann–Whitney U test because the data distribution was skewed.

Data are expressed as normalized means ± standard error of the mean (SEM), unless stated otherwise. The alpha level was set to 0.05 in all tests. All analyses were performed using IBM SPSS (version 29.02).

### 2.10. Graphics

Figures were made using Excel, PowerPoint, and Adobe for pdf.

## 3. Results

### 3.1. Subject Demographics

Demographics of the total group and responder subgroups per paradigm are presented in Table 2. Responders were defined by estimating the grand mean of all post-PAS measurements and dividing it by the baseline mean. A quotient > 1.0 was required for PAS-25 and <1.0 for PAS-10. However, since only three subjects qualified as LTD responders (post/pre mean ratio < 1.0), no statistical analysis was computed on that sub-sample. Instead, it turned out that in both PAS-10 paradigms, 10 versus 12 participants qualified as LTP responders (i.e., a post/pre MEP mean > 1.0), see Table 2.

### 3.2. No Significant Difference in RMT Between Test Sessions

To assess the within-subject test–retest validity between sessions, a repeated measures analysis of the RMT was performed. No significant difference was found F(3;39) = 1380, *p* = 0.263.

### 3.3. PAS-10 with 180 or 225 Paired Pulses Did Not Result in Decreased but Increased Corticospinal Excitation

**Figure 2:**
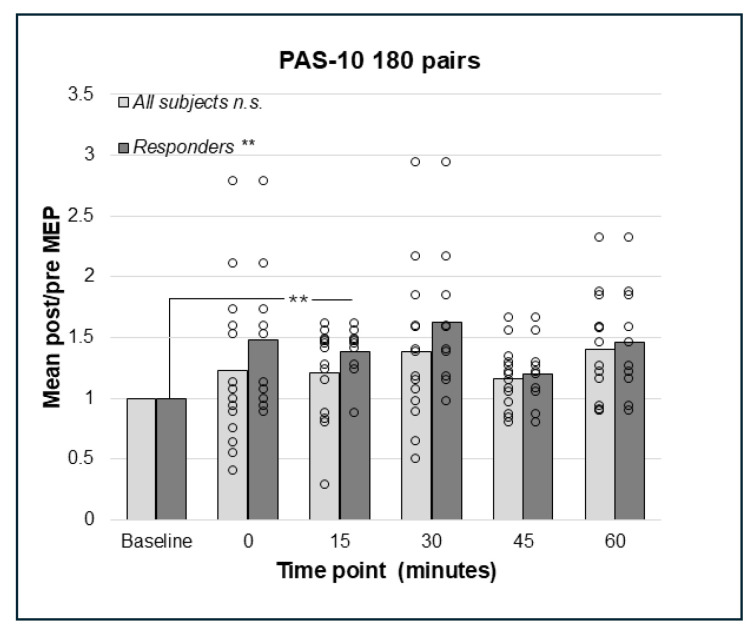
Relative change in MEP response from baseline (mean post/pre ratio = 1.0) to 0, 15, 30, 45, and 60 min post-PAS for PAS-10 with 180 PnS + TMS pairings; bars represent responders and non-responders. No significant effects of PAS-10 with 180 pairings were found in the whole sample. In responders (n = 10), a significant overall effect of PAS-10 180 pairs; *p* = 0.002 was found, and post hoc pairwise comparisons showed a significant different between baseline and 15 min; *p* = 0.004. ** = *p* < 0.01. n.s. = non-significant.

Repeated measures ANOVA did not detect any significant effects of PAS-10 with 180 pairings in the whole sample of subjects (N = 14), F(2.667;34.673) = 2.446, *p* = 0.087, Greenhouse–Geisser corrected (Figure 2).

**Figure 3:**
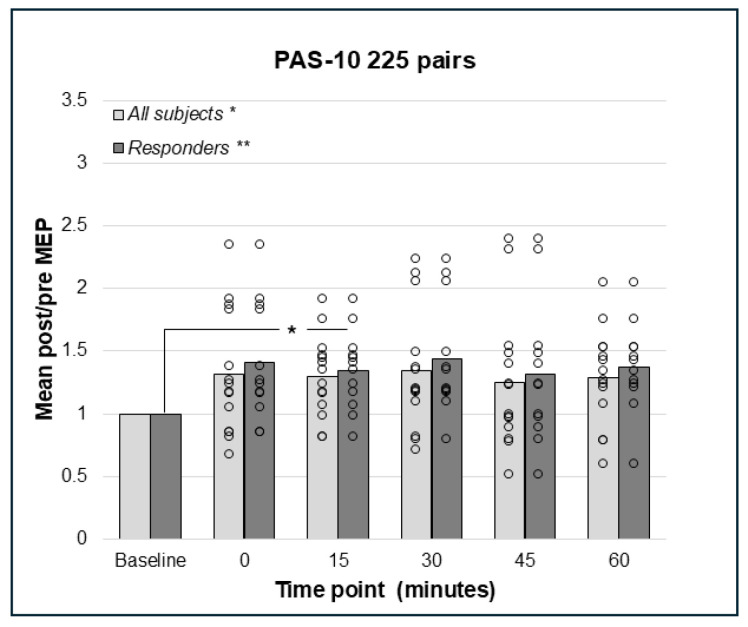
Relative change in MEP response from baseline (mean post/pre ratio = 1.0) to 0, 15, 30, 45, and 60 min post-PAS for PAS-10 with 225 PnS + TMS pairings; bars represent responders and non-responders. A significant effect for PAS-10 and 225 paired pulses was found in the whole sample; *p* = 0.046, but no pairwise differences post-PAS. In responders (n = 12), there was a significant overall effect; *p* = 0.014, and post hoc pair wise comparisons showed a significant effect comparing baseline to 15 min; *p* = 0.041. * = *p* < 0.05, ** = *p* < 0.01.

Repeated measures analysis for PAS-10 and 225 paired pulses identified a statistically significant effect in the whole sample (N = 14); F(5;65) = 2.41, *p* = 0.046, such that there was an increase in post-PAS MEP amplitude. However, pairwise comparisons did not detect any significant differences between baseline and post-PAS MEPs (Figure 3). Thus, both PAS-10 paradigms increased cortico-spinal excitability (see Figure 2 and Figure 3), and the analysis of responders (post/pre mean ratio > 1.0) resulted in 10 (PAS-10 180 pairs, Figure 2) and 12 (PAS-10 225 pairs, Figure 3) responders, respectively. Repeated measures ANOVA in responders detected a significant enhancing effect of PAS-10 180 pairs (n = 10); F(5;45) = 4.63, *p* = 0.002 at 15 min; *p* = 0.004 (Figure 2). Regarding PAS-10 225 pairs, there was a similar effect in responders (n = 12): F(5;55) = 3.15, *p* = 0.014, at 15 min; *p* = 0.041 (Figure 3).

### 3.4. PAS-25 with 180 and 225 Paired Pulses Resulted in a Significant Increase in the Corticospinal Excitation

**Figure 4:**
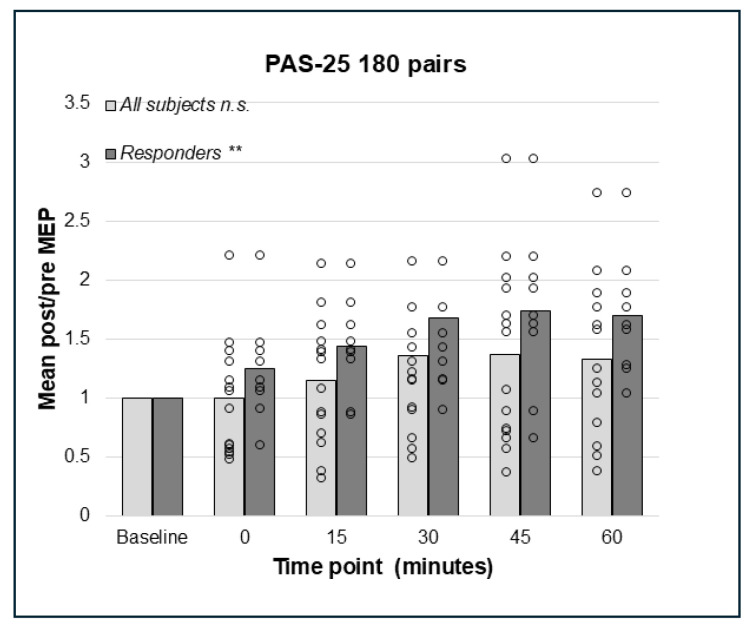
Relative change in MEP response from baseline (mean post/pre ratio = 1.0) to 0, 15, 30, 45, and 60 min post-PAS for PAS-25 with 180 PnS + TMS pairings; bars represent responders and non-responders. No significant effects of PAS-25 with 180 pairings were found in the whole sample. In responders (n = 9), a significant overall effect of PAS-10 180 pairs; *p* = 0.004 was found, but post hoc pairwise comparisons were not significant. ** = *p* < 0.01. n.s. = non-significant.

**Figure 5:**
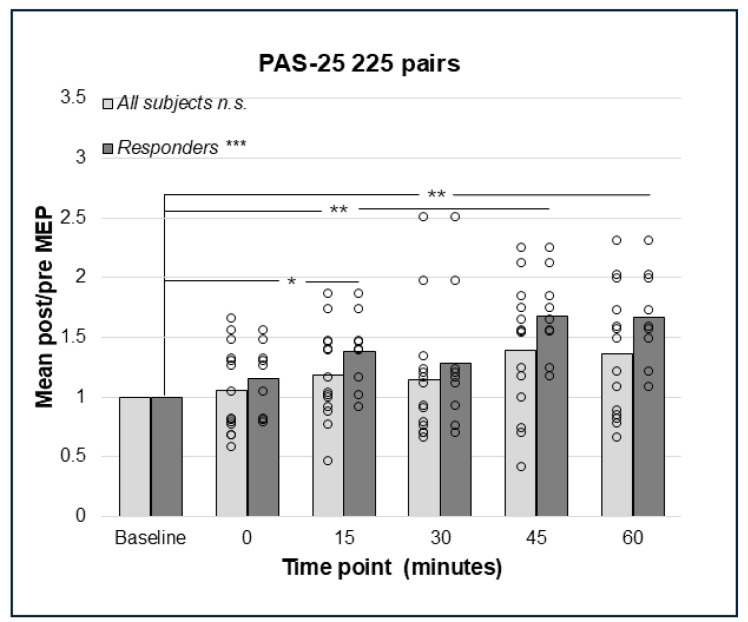
Relative change in MEP response from baseline (mean post/pre ratio = 1.0) to 0, 15, 30, 45, and 60 min post-PAS for PAS-25 with 225 PnS + TMS pairings; bars represent responders and non-responders. No significant effect for PAS-25 and 225 paired pulses was found in the whole sample. In responders (n = 9), there was a significant overall effect; *** *p* < 0.001, and post hoc pair wise comparisons showed a significant effect comparing baseline to 15 min; * *p* = 0.015, 45 min; ** *p* = 0.008 and 60 min; *p* = 0.008. n.s. = non-significant.

Repeated measures ANOVA did not detect a significant effect on the cortico-spinal excitability of PAS-25 with 180 pairings in the whole sample (N = 14), F(3.03;39.39) = 2.57, *p* = 0.067, Greenhouse–Geisser corrected results (Figure 4). However, the analyses of responders only (n = 9) using rmANOVA detected a significant effect, F(5;40) = 4.09, *p* = 0.004, indicating enhanced cortico-spinal excitability. Bonferroni-corrected pairwise comparisons did not detect any significant effect between pre- and post-PAS MEPs; however, there was a trend towards a difference between baseline and post-PAS MEPs at 60 min (*p* = 0.055, Figure 4). For PAS-25, with 225 pairs, data for post-PAS testing at post 0 min for the whole sample and 30 min for responders were not normally distributed. The Friedman test identified no significant effect of PAS-25 with 225 pairs in the whole sample (N = 14); Chi2(5) = 10.44, *p* = 0.064 (Figure 5); however, there was a significant increase in MEP amplitudes post-PAS in responders (n = 9); Chi2(5) = 21.51, *p* < 0.001. Wilcoxon signed rank test detected significant differences between baseline and post-PAS post/pre mean ratios at 15 min (*p* = 0.015), 45 min (*p* = 0.008), and 60 min (*p* = 0.008) in responders, see Figure 5.

**Figure 6:**
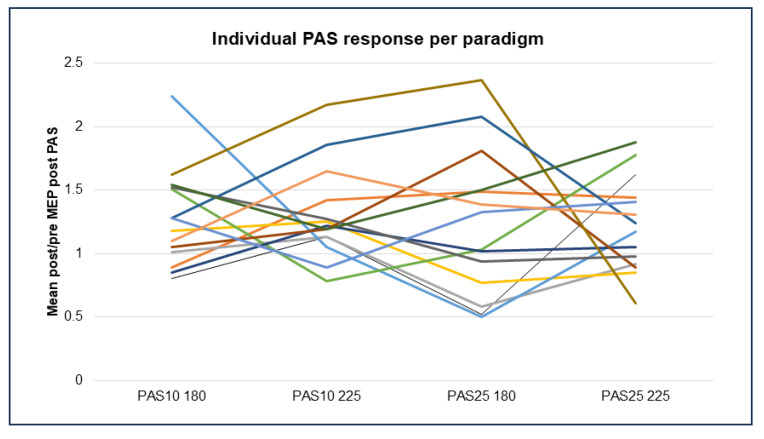
Individual PAS response per healthy participant, where each participant is signified by a different colored line (N = 14) in each of the four PAS paradigms, represented by mean change in MEP response, comparing baseline to all post-PAS time points. Abbreviations: PAS = paired associative stimulation and MEP = motor evoked potential.

Comparison of PAS outcomes between the four paradigms (PAS-10 and -25 with 180 versus 225 pairings) using paired samples *t*-tests detected no significant difference in mean post/pre ratios in the whole study group. This may at least partly be explained by the great variability in individual PAS responses between different PAS paradigms (see Figure 6).

### 3.5. Attentional Measure

There was no statistical difference in the means of calculated discrepancy between recited and delivered stimuli during intervention between the PAS-180 and 225 paradigms analyzed using the Mann–Whitney U test (U = 82.5, *p* = 0.475).

## 4. Discussion

This small randomized, double-blinded, crossover method study, aimed at evaluating four different PAS paradigms, two for assessing LTD (PAS-10 with 180 and 225 pairings) and two for assessing LTP (PAS-25 with 180 and 225 pairings) in healthy participants. The results demonstrated increased corticospinal excitability as indicative of LTP in all four PAS paradigms. As such, the PAS-10 protocol did not induce the expected decrease in corticospinal excitability, and there was large individual variability in response to all four paradigms. Notably, the PAS-225 protocol achieved significant PAS-25 (i.e., increased corticospinal excitability/LTP-like effects) at the group level of responders and yielded significant changes from baseline to post-PAS measures, whereas PAS-25 180 pairs did not produce a significant effect at post-PAS measures. This indicates that PAS-25 with 225 pairs is the most robust.

### 4.1. PAS-25 with 225 Pairings

PAS-25 with 225 pairings significantly increased corticospinal excitability in responders, and the pairwise comparisons identified significant differences between baseline and 15, 45, and 60 min post-PAS. The post-PAS excitability is rarely uniform, as illustrated by the lack of significance at 0 and 30 min post-PAS herein. Variability in post-PAS increases often occurs; for example, in Kaneko et al. (2024), healthy participants displayed an increased cortical excitability after PAS-25 with 180 pairings at post 0 and 15 min but not at 30 min post-PAS [32]. Bidirectional and non-linear plasticity may occur as PAS-induced plasticity depends on bidirectional homeostatic plasticity mechanisms in which the direction of PAS effect is dependent upon previous excitability as the brain has a built-in system to maintain an optimal range of excitability [33]. PAS mimics spike-timing-dependent plasticity (STDP) in which plasticity changes may be delayed due to required molecular mechanisms, such as NMDA receptor activation and AMPA receptor recruitment [34]. As such, Fransteva et al. (2008) using PAS-25 with 180 pairings found an increased cortical facilitation at 30 and 60 min but not at 0 and 15 min post-PAS [9]. In addition, differences in PAS effects between studies are likely due to a range of individual differences [35] and different stimulation parameters, such as stimulation intensity and the number of PAS pairings [36].

The PAS protocols with 225 pairings yielded lower variance, compared to the PAS-180 pairings. The duration of PAS with 225 pairings was 15 min, while 180 pairings took 30 min. Previous studies established greater facilitation using shorter protocols [28]. This aspect of shorter interventions is worth considering as the subject’s attentional level significantly influences PAS-induced plasticity [20]. Our results did not reveal a significant difference in attention levels between PAS protocols, yet participants frequently reported that the longer PAS-180 paradigm, compared to the PAS-225 paradigm, was particularly taxing. This difference in perception could explain the greater variability in the data from the PAS-180 group. It is also possible that the 225 pairings option yields a stronger corticospinal excitability due to more pulses than 180; as such, a dose-response PAS-25 study found that 270 pairings induced more robust LTP-like effects than 90 and 180 pairings [36].

Taken together, PAS-25 with 225 pairings was the most reliable and well-tolerated LTP protocol, and the results were in line with the a priori hypothesis of enhanced corticospinal excitability.

### 4.2. PAS-10 Increased Corticospinal Excitability

Surprisingly, the overall effect of the supposed LTD-inducing paradigms (PAS-10 with 180 and 225 pairs) resulted in a significant increase in excitability. Wischnewski and Schutter (2016) [21] evaluated the magnitude of PAS effects in 89 studies and found larger effects for PAS LTP compared to PAS LTD. In total, 22 studies on LTD were included in the meta-analysis, and the comparably low number of PAS LTD studies using the same stimulation parameters as the current study limits comparison. One stimulation parameter influencing corticospinal excitability is TMS intensity (i.e., sub- versus supra threshold intensities) during PAS stimulation.

As PAS mimics STDP, the level of postsynaptic depolarization regulates NMDAR activation and the likelihood of postsynaptic spiking [34]. In patch clamp studies, a shift from LTD to LTP appears with increasing levels of postsynaptic depolarization during presynaptic input [34]. Increasing TMS intensity leads to increased excitatory input to postsynaptic corticospinal neurons, increasing the likelihood for LTP with supra-threshold intensities [37]. Using sub-threshold (<100% of RMT) TMS stimulation during PAS results in decreased corticospinal excitability, i.e., LTD [38]. In here, the supra-threshold TMS intensity of 120% of RMT during PAS was used, and it is thus possible that the excitatory input to postsynaptic corticospinal neurons resulted in enhanced corticospinal excitement and, hence, the unexpected LTP-like effects of PAS-10.

Taken together, the PAS-10-induced increase in corticospinal excitability in the present study may be due to supra-threshold TMS intensity during PAS. In general, the PAS-10 paradigm appears more volatile than the PAS-25/LTP paradigms and is far less studied; thus, the relatively low number of small studies calls for studies with greater power to establish the TMS parameters that produce a robust PAS-10 effect.

### 4.3. Patterns of Response in the Different PAS Paradigms

It has been established that not all healthy individuals display changes in cortico-spinal excitability due to PAS. In the present study, this was the case for 9 of 14 participants as PAS-25 responders. Minkova and colleagues (2019) [39] used PAS-25, 180 pairings, and established a 61% response rate, and the resting-state connectivity of the sensorimotor network was positively correlated with PAS response in responders. No other baseline predictor (i.e., age, sleep quality, or cognitive impairment) was found [39]. Lopez-Alfonzo and colleagues (2014) used PAS LTP (PAS-25, 200 pairings, response = grand mean > 1.0), resulting in response rates of 54% [35]. No predictive relationship of baseline measures (the time of day, age, stimulation intensity/resting motor thresholds, or baseline short-interval cortical inhibition) and PAS response were identified. Studies have established effects of attention [20], age [40], and the time of day for PAS facilitation [40]; however, no single variable seems sufficient to reliably predict PAS response.

Herein, a different pattern of responses was noted than expected as all PAS paradigms tended to be facilitatory. More individuals responded inhibitory to the PAS-25 paradigms than the PAS-10 paradigms (Figure 6). The PAS-25 180 pairings yielded the largest variance in responses. The PAS-10 with 225 pairings resulted in facilitation in 12 out of 14 patients, thus providing a robust increase in excitability post-PAS in most subjects but with a greater variance than PAS-25 with 225 pairings. Of note is that the test–retest reliability of PAS-25 has previously been demonstrated to be surprisingly low [41]. The present study suggests that the responses to different PAS paradigms are individual, thus blunting significant findings using group-based analysis.

## 5. Limitations

This study is primarily limited by the number of study participants (N = 14), especially since 14 to 29% of participants were non-responders in the different PAS paradigms. However, the majority of previous PAS studies (see [21] for a comprehensive review) tend to be in the lower range of participants (≤14 participants per treatment group). The number of non-responders in this study is in comparison with other PAS studies. Despite this, the limited participant number in this study contributes to the variability in responses between paradigms and limits the interpretation of the results; thus, larger trials are needed to establish the effect of the current study.

## 6. Conclusions and Future Direction

In this methodological study, PAS-25 delivering 225 paired pulses at 0.25 Hz, produced a significant enhancement of motor cortico-spinal excitability lasting up to 60 min in healthy controls, while supposedly PAS LTD-inducing protocols failed to decrease excitability. The small study sample limits the interpretation of the unexpected PAS-10 data; however, supra-threshold TMS intensity during PAS may explain the LTP-like effects. As there is no consensus as to what threshold is needed for LTD-like effects, additional comparative supra- versus sub-threshold studies are warranted. In general, there is a lack of adequately powered PAS LTD studies; thus, larger studies are required to elucidate the effects and underlying mechanisms of PAS LTD. The application of PAS-25, with 225 pairings, at 0.25 Hz is reliable, time-efficient, and user-friendly, promoting future studies of human cortical plasticity.

## Figures and Tables

**Table 1:** Stimulation parameters of the four PAS paradigms.

	Phase 1		Phase 2	
PAS paradigm	PAS-10 (180)	PAS-25 (180)	PAS-10 (225)	PAS-25 (225)
Inter-stimulus interval (PnS to TMS)	10 ms	25 ms	10 ms	25 ms
Number of PnS + TMS pairs	180	180	225	225
Inter-pair interval	10 s (0.1 Hz)	10 s (0.1 Hz)	4 s (0.25 Hz)	4 s (0.25 Hz)

**Table 2:** Demographics in all participants and in responders per PAS paradigm.

Group	Full Participation Group	Mean Post/Pre > 1.0	Mean Post/Pre > 1.0	Mean Post/Pre > 1.0	Mean Post/Pre > 1.0
Paradigm	All	PAS-10 180 pairs	PAS-10 225 pairs	PAS-25 180 pairs	PAS-25 225 pairs
Participants (#)	14	10	12	9	9
Females/males	6/8	2/8	6/6	4/5	4/5
Mean (±s.d.) age years baseline	32.29 (7.72)	34.90 (6.37)	31.58 (8.10)	33.78 (7.16)	33.00 (8.03)
Mean (±s.d.) height (cm) baseline	172 (12.70)	174.00 (12.95)	172.92 (11.60)	171.56 (15.57)	170.89 (14.49)
Mean (±s.d.) AUDITc baseline	4 (1.3)	n.a.	n.a.	n.a.	n.a.
Mean (±s.d.) RMT %	39.79 (6.07)	39.80 (5.79)	40.25 (7.21)	39.89 (7.54)	39.67 (6.71)
Mean (±s.d.) stimulus intensity %	n.a.	50.40 (8:41)	48.25 (10.36)	52.00 (12.51)	47.67 (10.38)

Abbreviations: RMT = resting motor threshold, AUDITc = the alcohol use disorder identification test c. s.d. = standard deviation.

## Data Availability

Data are stored at the Gothenburg University’s environment for Secure storage service (information class 3).

## Abbreviations

The following abbreviations are used in this manuscript:

EMG	Electromyographic
M1	Primary motor cortex
LTP	Long-term potentiation
LTD	Long-term depression
rTMS	Repetitive transcranial magnetic stimulation
PAS	Paired associative stimulation
PnS	Peripheral nerve stimulation
RMT	Resting motor threshold
STDP	Spike-timing-dependent plasticity
